# An endangered West African rattan palm: *Eremospatha dransfieldii*

**DOI:** 10.3897/BDJ.5.e11176

**Published:** 2017-01-13

**Authors:** Ariane Cosiaux, Lauren M. Gardiner, Doudjo N. Ouattara, Fred W. Stauffer, Bonaventure Sonké, Thomas L.P. Couvreur

**Affiliations:** 1 Institut de Recherche pour le Développent (IRD), Yaoundé, Cameroon; 2 Plant Systematic and Ecology Laboratory, Higher Teacher’s Training College, University of Yaoundé I, Yaoundé, Cameroon; 3 Royal Botanic Gardens, Kew, London, United Kingdom; 4 Université de Genève, Genève, Switzerland; 5 Conservatoire et Jardin botaniques de la Ville de Genève, Chambésy, Switzerland; 6 Conservatoire et jardin botaniques, Chambésy, Switzerland; 7 Institut de Recherche pour le Développement (IRD), Montpellier, France

**Keywords:** Endangered species, habitat loss, over-harvesting, rattan palm, West Africa, Ghana, Côte d'Ivoire, Sierra Leone

## Species information

Scientific name: Eremospatha dransfieldii

Species authority: Sunderl.

Common names: Rattan (English), Rotin (French); Sierra Leone: balu (Kono); mbalu (def. -ui) (Mende); Côte d’Ivoire: kpè-pun (Attié); tami (Dyula); kou gnain (Gouro); niböigain (Wè): Ghana: mfia (Twi) (Sunderland et al. 2005).

Kingdom: PLANTAE

Phylum: TRACHEOPHYTA

Class: LILIOPSIDA

Order: ARECALES

Family: PALMAE

Taxonomic notes: Eremospatha dransfieldii (Sunderland 2003) has been shown to be a sister species to E. cabrae (De. Wild. & Th. Dur.) De Wild. from cenrtral Africa based on phylogenetic analyses of molecular sequence data (Faye et al. 2014, Faye et al. 2016).

**Figure 1.**
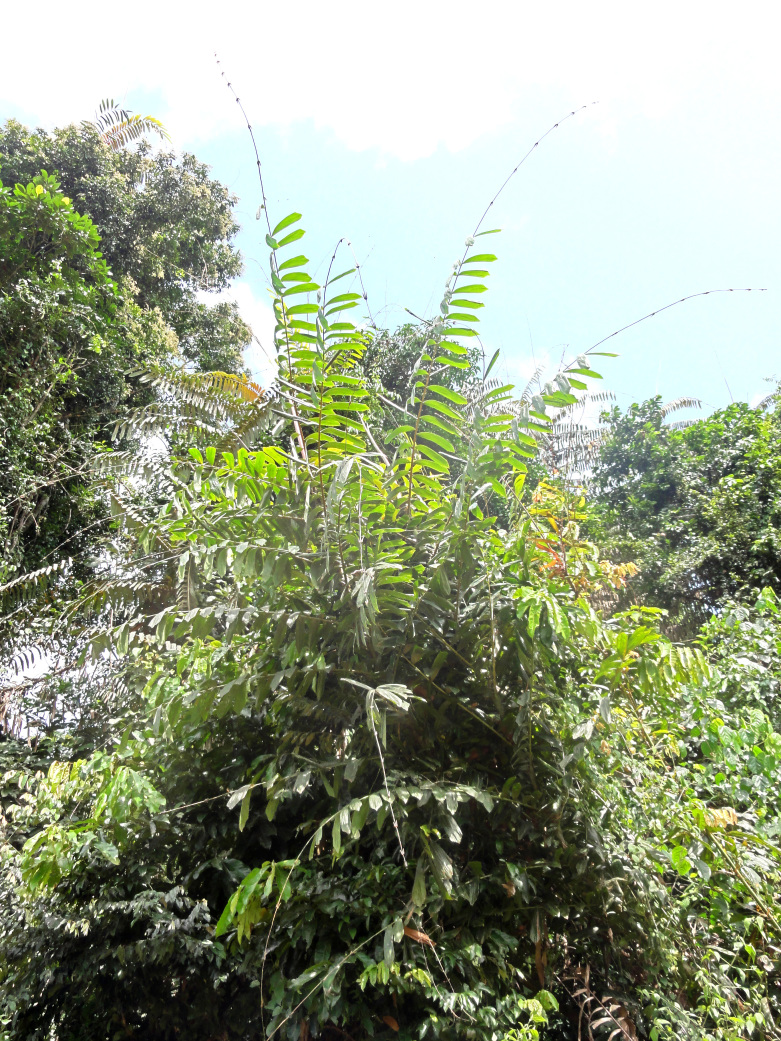
*Eremospatha dransfieldii* in the "Forêt Classée de N'zodji", Côte d'Ivoire [Photo Credit: Doudjo Ouattara, 2016]

**Figure 2.**
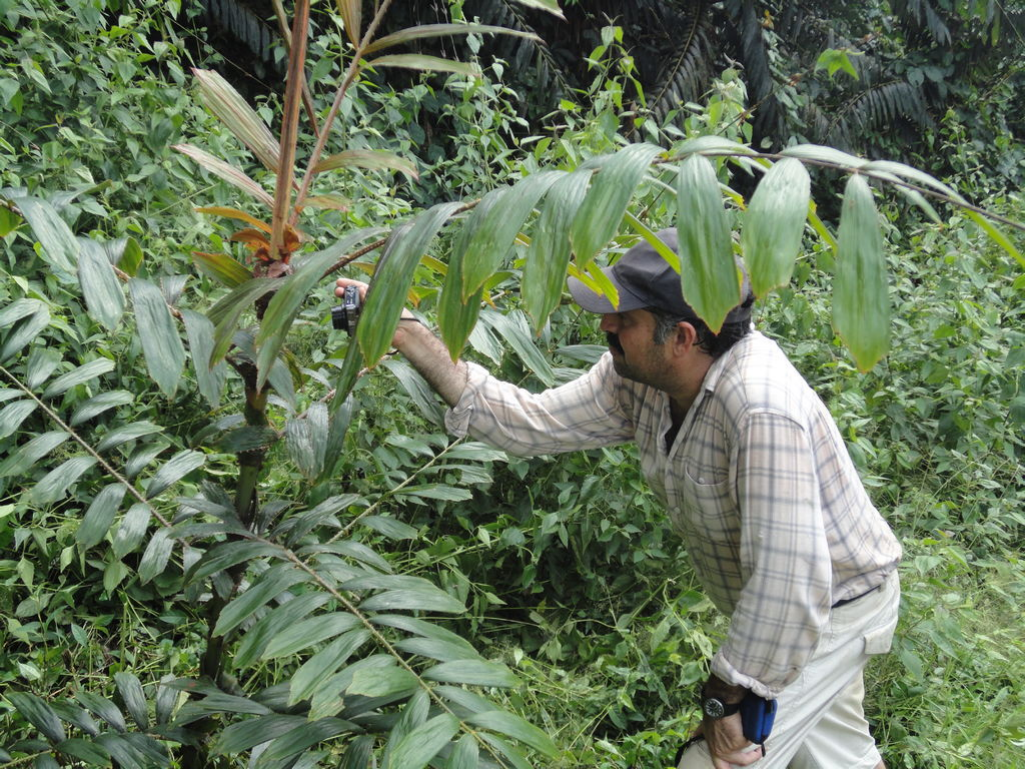
Dr. Fred Stauffer photographing *Eremospatha dransfieldii* in the "Forêt classée de N'zodji", Côte d'Ivoire [Photo Credit: Doudjo Ouattara, 2016]

Figure(s) or Photo(s): Figs 1, 2

Region for assessment: Western Africa

## Geographic range

Biogeographic realm: Afrotropical

Countries: Sierra Leone

**Figure 3.**
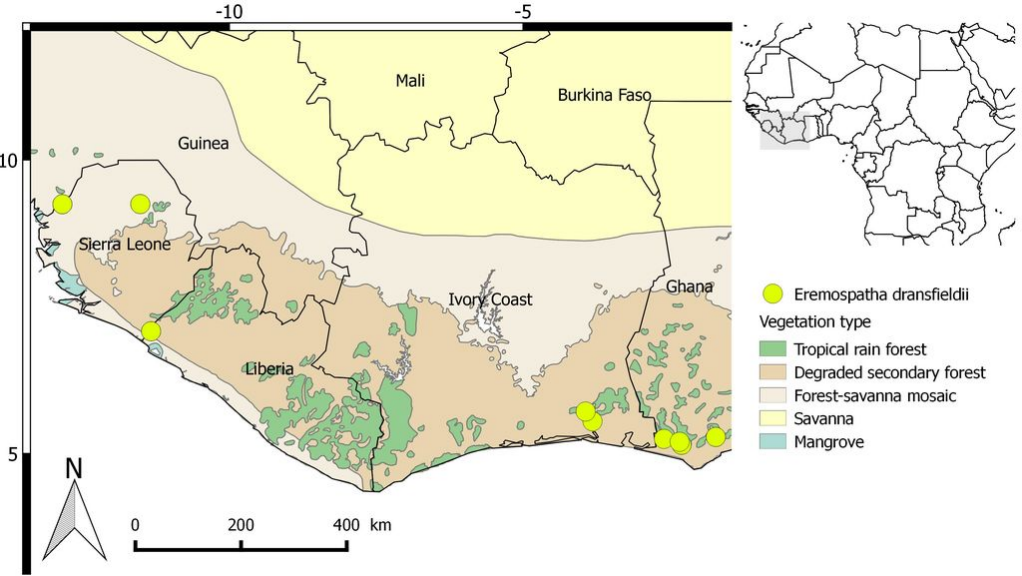
Distribution map of *Eremospatha dransfieldii*.

Map of records (image): Fig. 3

Map of records (Google Earth): 

Basis of EOO and AOO: Observed

Basis (narrative): The Extent of Occurence (EOO) was calculated using 20 georeferenced herbarium specimens found in the RAINBIO database (Dauby et al. 2016). Within the EOO, the Area of Occupancy (AOO) was calculated as the sum of occupied cells after superimposing a grid with cells of 2x2 km. Both parameters were estimated using the web service GeoCAT (Bachman et al. 2011).

Min Elevation/Depth (m): 100

Max Elevation/Depth (m): 200

Range description: Eremospatha dransfieldii is only found in the south-western region of Ghana and south-eastern part of Côte d'Ivoire, with two specimens collected from Sierra Leone. It is reported from 100 to 200 meters above sea level (a.s.l.). In Ghana, the species is restricted to forest reserves and conservation areas of the Western Region. In Côte d'Ivoire it has only been recently discovered, in the "Forêt classée N'zodji" (S. da Giau, D. Ouattara and F. Stauffer pers. comm. 2016). The EOO of the species is large (251 334 km²). Within the EOO, the AOO is restricted to only 40 km². Rattans are generally under-collected by researchers because they are spiny climbers and hard to collect so the low AOO may be an artefact linked to this. However, this rattan palm appears to be confined to forests with high rainfall (Sunderland 2007, Sunderland 2012), so the low AOO is likely to be a reflection of the species' true distribution.

## Extent of occurrence

EOO (km2): 251﻿334

Trend: Unknown

Justification for trend: There is no current available data about the trend of the Extent Of Occurrence.

Causes ceased?: Unknown

Causes understood?: Unknown

Causes reversible?: Unknown

Extreme fluctuations?: Unknown

## Area of occupancy

Trend: Unknown

Justification for trend: There is no current available data about the trend of the Area Of Occupancy.

Causes ceased?: Unknown

Causes understood?: Unknown

Causes reversible?: Unknown

Extreme fluctuations?: Unknown

AOO (km2): 40

## Locations

Number of locations: 

Trend: Unknown

Extreme fluctuations?: No

## Population

Trend: Decline (inferred)

Basis for decline: (c) a decline in area of occupancy, extent of occurrence and/or quality of habitat

Causes ceased?: No

Causes understood?: Yes

Causes reversible?: Yes

Extreme fluctuations?: Unknown

Population Information (Narrative): The number of mature individuals is not known. Although the species is poorly known, it is likely to be very rare due to the limited amount of forest habitat remaining across its range. The known sub-populations are isolated by large distances and occur in small patches of forest surrounded by a matrix of unsuitable habitat. Recent surveys suggest there are only a small number of individuals at some sites e.g. Forêt Classée de N’zodji, Côte d'Ivoire, or even no individuals at all remaining in some places e.g. Ankasa Natural Reserve, Ghana (F. Stauffer, pers. comm. 2016). Due to the fragmentation of the forest habitat it is likely that most of the individuals of this species persist in isolated patches with low viability and therefore it should be considered severely fragmented. Moreover, the sub-populations are known to be facing habitat loss and over-harvesting, as a result we can infer that the population is currently likely to be decreasing.

## Subpopulations

Trend: 

Extreme fluctuations?: No

Severe fragmentation?: No

## Habitat

System: Terrestrial

Habitat specialist: Unknown

Habitat (narrative): This species only occurs in moist evergreen forests with rainfall higher than 2000 mm/year, where it prefers to grow in tree-fall gaps and forest margins (Sunderland 2012, Sunderland 2007).

Trend in extent, area or quality?: Decline (observed)

Justification for trend: This rattan does not occur in agro-ecosystems and it is highly threatened by habitat loss in Ghana and Côte d'Ivoire (S. da Giau, D. Ouattara and F. Stauffer pers. comm. 2016) - this rattan is subject to a continuing decline of the extent of its habitat.

### Habitat

Habitat importance: Major Importance

Habitats: 1. Forest

## Ecology

Generation length (yr): 

Dependency of single sp?: No

Ecology and traits (narrative): Eremospatha dransfieldii is a robust rattan palm, with multiple stems and climbing to 40 m in length (Sunderland et al. 2005, Sunderland 2012, Sunderland 2007, Sunderland 2003).

## Threats

Justification for threats: The major threats to this species are habitat loss due to forest conversion to agriculture, and over-harvesting of the stems for the rattan products (D. Ouattara and F. Stauffer, pers. comm. 2016). In Côte d'Ivoire, this species is highly threatened; the only forest where the species has been observed is currently being cut to open up new fields. The stems of this rattan are over-harvested in the area, which could soon lead to the disappearance of this species in the country. The same threats are observed in Ghana. Data are currently absent regarding the threats to this species in Sierra Leone, but we infer that the species is likely to be threatened by habitat loss there too.

### Threats

Threat type: Ongoing

Threats: 2. Agriculture & aquaculture

## Conservation

Justification for conservation actions: This rattan palm appears to be highly threatened by habitat loss and over-harvesting and it is not well represented inside protected areas.Based on known herbarium records, this species presents a large geographic distribution (ie. its EOO is large), but its AOO is limited to just 40 km². Although this may in part be a result of botanical under-collecting, the specialized habitat of this species suggests that it only grows in moist evergreen forests with high rainfall and that it has a genuinely restricted distribution. Based on this, and the ongoing habitat loss, fragmented distribution, and overharvesting of the species, Eremospatha dransfieldii is assessed as Endangered based on criteron B2ab(iii), according to the IUCN Red List Categories and Criteria (ver 3.1). Indeed, the assessment is based on B2 (small AOO), a - severe fragmentation, b - continuing decline in suitable habitat area.Conservation measures are urgently needed to protect the habitat of this species and to control the unsustainable harvest of the stems. A promising solution might be the sustainable cultivation of rattans (Sunderland and Nkefor 2002) to avoid the exploitation of wild populations. The only place where the species is represented in a protected area is in the Ankasa Conservation Area in Ghana.

### Conservation actions

Conservation action type: In Place

Conservation actions: 1. Land/water protection

## Other

Justification for use and trade: This rattan is traded in local markets, in Ghana and Côte d'Ivoire. The species is mainly used for furniture frames and coarse basketry (Sunderland 2007). In Ghana, most people are not able to differentiate Eremospatha dransfieldii from Eremospatha macrocarpa which are used for the same purposes. The canes are usually longitudinally split into several ribbons, which are used to attach the frames of furniture, used as ropes for thatching, for making baskets and sieves and to make traps (Ouattara et al. 2015). As with most African rattan species, there is inadequate information on the international trade, but it is likely to be negligible, although this rattan may have been among the species exported from Ghana to the United Kingdom in the period between the two World Wars (PROTA 2015, http://uses.plantnet-project.org/fr/Eremospatha_dransfieldii_(PROTA)).

### Use and trade

Use type: National

Use and trade: 9. Construction or structural materials
